# The Frequency of Occurrence of Strabismus in Infants Post Lensectomy

**DOI:** 10.7759/cureus.73024

**Published:** 2024-11-04

**Authors:** Jonas Akambase, Michael Barkley, Richa Sharma, Ann Webber, Shuan Dai

**Affiliations:** 1 Ophthalmology, Queensland Children's Hospital, Brisbane, AUS

**Keywords:** bilateral, cataract, esotropia, exotropia, infant, interocular, lensectomy, strabismus, unilateral, visual acuity

## Abstract

Purpose

To identify and characterize incident cases of strabismus and interocular visual acuity among infants post lensectomy for congenital cataract.

Method

This was a single-centre retrospective chart review of all children aged less than 12 months who underwent lensectomy from 1st January 2014 to 1st January 2021. Cases were identified from theatre coding and electronic medical records. Cases with strabismus prior to cataract surgery were excluded.

Results

Seventy-five children (114 aphakic eyes) were included, 36 (48%) had unilateral cataract surgery while 39 (52%) had bilateral. The mean age at the time of cataract surgery was 3.0±2.5 months (range 1 - 10 months). The mean follow-up period was 41.2±22.8 months (range 2 -72 months). Nineteen out of 75 patients (25%) developed strabismus, most of whom (18 cases) had unilateral surgery. Out of the 19 strabismus cases recorded, esotropia was reported in 74% of the cases, followed by exotropia -16%, while esotropia associated with dissociated vertical deviation (DVD) and esotropia associated with inferior oblique each made up 5% of the population. Most children who developed strabismus [17 patients (89.5%)] had lensectomy prior to 6 months of age. A significant difference of 0.65 logMAR was recorded in the interocular visual acuity (IOVA) difference between the strabismic and non-strabismic groups, and the unilateral and bilateral lensectomy groups.

Conclusion

Strabismus, most commonly esotropia, is common after congenital lensectomy, particularly in those children who underwent unilateral cataract surgery, and in those less than 6 months of age. Monitoring IOVA and strabismus incidence post-cataract surgery is crucial for early intervention and amblyopia prevention.

## Introduction

Strabismus is a well-documented complication that can occur in aphakic infants following lensectomy for congenital cataracts. The incidence of strabismus associated with congenital cataracts has been reported to range from 17.2% - 63.9% before and after cataract surgery. Prior reports have found a strong correlation between cataract surgery and strabismus in children, with main risk factors including laterality of the cataract (unilateral or bilateral), persistent foetal vasculature (PFV), poor final visual acuity, and young age at surgery. Strabismus occurs more frequently in patients with unilateral cataracts than those with bilateral cataracts, and this is due to poor visual acuity that limits the sensory fusion of retinal images [1 - 7].

In this study, we determine the prevalence of strabismus developing in aphakic children following surgery for unilateral or bilateral cataracts. The influence of age at surgery and visual outcome on the frequency of strabismus was assessed.

The group of children who were aphakic post-surgery was corrected with a custom aspheric rigid gas permeable (RGP) contact lens (CL), and those aged < 2 years were deemed unsuitable for intraocular lens (IOL) implant due to the rapid change in ocular biometrics in the first 12 months of life and a high number of complications reported [8 - 11].

This article was previously presented as a meeting abstract at the 2022 and 2023 RANZCO Annual Scientific Meeting on October 28, 2022 and October 20, 2023, respectively.

## Materials and methods

A single centre retrospective chart review of all children aged less than 12 months who underwent lensectomy for congenital cataracts from 1st January 2014 to 1st January 2021 whose subsequent aphakia was corrected with custom aspheric rigid gas permeable contact lens (RGP CL). This study was conducted at the Queensland Children's Hospital in Australia. Cases were identified from theatre coding and electronic medical records. Cases with strabismus prior to cataract surgery, or a history of ocular trauma, prematurity, and co-existent metabolic, genetic, or other systemic disorders were excluded. Lensectomies were performed by different surgeons either by pars plana approach or via clear cornea. Ocular alignments were taken using the Hirshberg and Krimsky test while patients were in contact lenses. The visual acuity before surgery was assessed using either F&F (fix and follow) or CSM (central, steady, and maintained), and the best visual acuity (VA) for the last follow-up after cataract surgery was recorded using Lea matching and Snellen. All visual acuity was converted to logMAR. All VA that were recorded as Hand Motions (HM), Fix and Follow (F&F), Central, Steady, and Maintained (CSM), No Light Perception (NLP), Counting Fingers (CF) were converted to numeric values as 2.3 logMAR, 2.3 logMAR, 0.3 logMAR, 2.6 logMAR, and 1.9 logMAR, respectively, using the conversion table described in Arnold (2021) [11].

Statistical analysis

All statistical analyses were performed using Excel (Microsoft Corporation, Redmond, USA). A two-sample test was employed to compare continuous variables such as age at surgery between the two groups. A value less than 0.05 was considered significant. Statistically, we sought to find the hypothesized mean difference between the visual acuity of children who had unilateral cataract surgery and those who had bilateral cataract surgery. After cataract surgery, some children developed strabismus and others did not, as a result, we statistically compared the post-operative mean visual acuity of those who developed strabismus and those who did not develop strabismus. In addition, among those who had unilateral cataract surgery, we statistically compared the mean visual acuity of those who developed strabismus and those who did not. 

Mathematical analysis

The interocular visual acuity (IOVA) difference was investigated among the strabismic and non-strabismic group, and the unilateral strabismic and unilateral nonstrabismic groups, and this was achieved by mathematically finding the difference in the visual acuity between the right and left eye of each participant in each group. The mean difference in IOVA between various groups was achieved by mathematically finding the difference between the average IOVA of the groups to be compared. 

Ethical approval was obtained from the Human Research Ethics Committee (HREC)​​​​​​,​ Queensland Children’s Hospital, and the study conducted adhered to the Declaration of Helsinki.

## Results

Seventy-five children (114 aphakic eyes) were included in the study, 36 (48%) children had unilateral lensectomy while 39 children (52%) had bilateral lensectomy. The mean age at the time of lensectomy was 3.0 ± 2.5 months (range 1 - 10 months). The mean follow-up period was 41.2 ± 22.8 months (range 2 -72 months), and the mean age at the last follow-up was 38.7 ± 20.0 months (range 4 - 73 months) and 45.9±23.4 months (range 8 - 76 months) for those who had unilateral lensectomy and for those who had bilateral lensectomy, respectively. The mean pre-operative visual acuity (VA) of 2.3 logMAR±0.1 (2.3-2.5) for both those who had bilateral lensectomy and those who had unilateral lensectomy (Table 1).

**Table 1:** Summary of patients demographics M - male ; F - female

Variables	All cases	Unilateral	Bilateral
Number patients (Number eyes)	75 (114)	36 (48%)	39 (52%)
Gender (M:F)	31:44	13:23	17:22
Mean age at time of surgery (Range/months)	3.0 ±2.5 (1–10)	2.2 ± 0.8 (1 –5)	2.6 ± 1.1 (1 – 10)
Mean follow up (Range/months)	41.2±22.8 (2 –72)	36.2±20.6 (2 – 70)	43.4±23.8 (5 – 72)
Mean age at last visit (Range/months)	42.6 ± 21.9 (4 – 76)	38.7 ± 20.0 (4 – 73)	45.9±23.4 (8 – 76)
Mean preoperative visual acuity/ LogMAR (Range)	2.3±0.1 (2.3-2.5)	2.3±0.1 (2.3-2.5)	2.3±0.1 (2.3-2.5)

The post-operative best corrected visual acuity (BCVA) was significantly different (p =0.02; <0.05) between the unilateral and bilateral lensectomy groups with mean VA of 0.4 logMAR ± 0.3 for the bilateral group and 0.7 logMAR ± 0.5 for the unilateral group. Among the unilateral lensectomy group, the VA for those who developed strabismus when compared with those who did not develop strabismus was reported to be 1.0 logMAR±0.9 (0.0 - 2.3) for the unilateral strabismic group and 0.7 logMAR±0.9 (0.0 - 2.5) for the unilateral non-strabismic group. This however indicated no significant difference between these two groups. In addition, the mean interocular visual acuity difference was almost similar for these two groups (1.0 logMAR±0.8 versus 0.9 logMAR±0.8, respectively). The BCVA was similar in the strabismic and non-strabismic (p=0.22; <0.0 5) aphakic eye. The mean interocular difference in VA was 0.32 logMAR ± 0.58 and 0.97 logMAR ±0.81 for the non-strabismic group and strabismic group respectively, and the mathematical difference (0.65 logMAR) between the mean IOVA of these two groups was considered significant (Table 2). 

**Table 2:** Mean post-operative visual acuity (VA), comparing bilateral vs unilateral lensectomy, and mean interocular visual acuity difference (IOVA), comparing non-strabismic vs strabismic group, unilateral strabismic vs unilateral non-strabismic, unilateral vs bilateral lensectomy groups. VA - visual acuity; IOVA - interocular visual acuity difference. All visual acuity was converted to logMAR. All VA that were recorded as Hand Motions (HM), Fix and Follow (F&F), Central, Steady, and Maintained (CSM), No Light Perception (NLP), Counting Fingers (CF) were converted to numeric values as 2.3 logMAR, 2.3 logMAR, 0.3 logMAR, 2.6 logMAR, and 1.9 logMAR, respectively, using the conversion table described in Arnold (2021) [11].

Variables	Post-operative								
	Unilateral	Bilateral	P-value	Strabismic	Non-strabismic	P-value	Unilateral strabismic	Unilateral non-strabismic	P-value
VA / LogMAR (Range)	0.7±0.5 (0.1 – 1.8)	0.4±0.3(0.1 – 1.3)	0.02	0.7±0.5 (0.3 – 1.8)	0.5±0.4 (0.1 – 1.4)	0.22	1.0±0.9 (0.0 – 2.3)	0.7±0.9 (0.0 – 2.5)	0.36
IOVA / LogMAR	0.9±0.8	0.1±0.2	NA	1.0 ±0.8	0.3 ±0.6	NA	1.0 ±0.8	0.9 ±0.8	NA

As shown in Table 3, 19 out of 75 patients developed strabismus, most of whom had unilateral aphakia (18 of 19 cases). Strabismus developed in 50% of unilateral (18 of 36) and 2.6% of bilateral cases (1 of 39). Out of the 19 strabismus cases recorded, esotropia was reported in 74% of the cases, followed by exotropia in 16%, while esotropia associated with dissociated vertical deviation (DVD) and esotropia associated with inferior oblique each made up 5% of the population.

**Table 3:** The frequency of occurrence of strabismus in infants post cataract surgery. DVD - dissociated vertical deviation; IOOA - inferior oblique overaction

Strabismus type	Unilateral < 12 months No. of cases	Bilateral < 12months No. of cases	Total
Esotropia	13	1	14 (74%)
Exotropia	3	0	3 (16%)
Esotropia with DVD	1	0	1 (5%)
Esotropia with IOOA	1	0	1 (5%)
Total No. Strabismus cases post lensectomy	18(50%)	1(2.6%)	19

More children who developed strabismus (17 patients (89.5%)) had lensectomy prior to 6 months of age, meanwhile, strabismus developed less frequently in those who had lensectomy after 6 months of age (two patients (10.5%)) (Table 4).

**Table 4:** Prevalence of strabismus in different age groups post lensectomy.

Age at Surgery / months	No of cases (percentage)
< 6	17 (89.5%)
>6 <12	2 (10.5%)

## Discussion

Strabismus is well known to be associated with congenital cataracts and post-cataract surgery in children. The main purpose of this study is to compare the prevalence of strabismus developing in children undergoing lensectomy for unilateral or bilateral cataracts and as well evaluate the frequency of strabismus according to age at surgery and visual outcome. The post-operative best-corrected visual acuity (BCVA) with contact lens (CL) for the 36 children who had unilateral lensectomy and 39 who had bilateral lensectomy in our study was significantly different. The BCVA for the unilateral group was poorer than the group that underwent bilateral lensectomy: a big challenge for those that have unilateral aphakia and a normal fellow eye are highly susceptible to dense amblyopia and must undertake intense occlusion to promote visual neurodevelopment. This is not something that the bilateral visual pathway has to contend with.

Similarly, in a study conducted by Repka et al. (2019) [1], the mean visual acuity was 0.30 logMAR (about 20/40) in 153 bilateral pseudophakic eyes, 0.49 logMAR (about 20/63) in 141 unilateral pseudophakic eyes, 0.47 logMAR (about 20/63) in 21 bilateral aphakic eyes, and 0.61 logMAR (about 20/80) in 17 unilateral aphakic eyes. In the Pediatric Eye Disease Investigator Group (PEDIG) study, there was a slight difference in VA for both the bilateral and unilateral pseudophakic eyes, also there was a difference in VA among the bilateral and unilateral aphakic eyes, but this was not statistically confirmed [1]. Our study is the first to include interocular visual acuity differences among strabismic and non-strabismic aphakic eyes. In this study, we reported a significant difference in the interocular visual acuity difference between the strabismic group and the non-strabismic group. This is an indication that the most likely factor that contributed to the strabismus is sensory amblyopia. However, the IOVA did not differ among the unilateral cases between those who did or did not develop subsequent strabismus. In addition, our study demonstrated a significant difference in the IOVA difference between bilateral and unilateral lensectomy groups. This explains that the unilateral lensectomy group is more likely to develop amblyopia compared to the bilateral.

In this research we demonstrated that 56 out of 75 children who did not develop strabismus after lensectomy had better vision in their aphakic eye than the 19 children who developed strabismus post lensectomy, however, the difference was not statistically significant. Likewise, Weisberg et al., reported that a larger number of pseudophakic patients with strabismus had poorer vision than those without strabismus, and poor visual acuity contributed to the strabismus [2]. Weisberg et al. included children with cataracts who had already developed strabismus, whereas, in our study, we exempted children who had already developed strabismus.

We found the prevalence of strabismus was higher in the unilateral lensectomy group than in the bilateral group (50% vs 2.6%). David et al reported 63.9% of strabismus cases in the unilateral lensectomy group. So, only having one strabismus in the bilateral group in this study is very good - 2.6% compared to nearly 30% in David et al. [3]. The group that had unilateral lensectomy was also found to record a higher number of strabismus cases (29.5%) than the group that had bilateral lensectomy (17.2%) in a study conducted by Lee et al. [4]. Park et al recorded a high number of strabismus cases in a group of children with bilateral lensectomy, however, their study did not include unilateral lensectomy [6]. The Infant Aphakia Treatment Study (IATS) reported strabismus to be prevalent in the group that underwent unilateral lensectomy, however, this study did not involve children who underwent bilateral lensectomy [5]. The IATS is a multicenter study while our study is a single-centre study. The aim of IATS is to compare the visual outcomes and complications of the correction of aphakia using a contact lens to those when an IOL is placed primarily after monocular lensectomy in infants aged 1 to 6 months [10].

Our study indicated that children operated on at a younger age recorded a higher rate of strabismus than children who were operated on at an older age (age at surgery <6 months vs >6 months, respectively). Likewise, David et al demonstrated that children who had lensectomy at a median age of 25.9 months recorded a higher number of strabismus cases than those who had lensectomy at a median age of 52.7 months [3]. Park et al also reported that 51.6% of children who had lensectomy in the first year of life developed strabismus [6]. Lee et al found that an age of less than a year at the time of surgery was related to the onset of strabismus in bilateral cataracts, but not in unilateral cases [4]. Conversely, IATS in their study revealed that children <49 days at the time of surgery reported significantly less number of strabismus cases (58.0%) as compared with children operated at age ≥49 days (80%)5. The reason IATS may have a different outcome when compared to our study is because the IATS study included children with pre-existing strabismus, meanwhile our study exempted children with pre-existing strabismus. Further, the children recruited for the IATS study were within the age range of 28 days to 7 months whereas our study included children within the age range of 1 month to 10 months. Moreover, our categorization of younger and older children differs from that of IATS: unlike IATS, which grouped children under 49 days as younger age children and those older than or equal to 49 days old as older age children, we grouped children under 6 months as younger age children and those older than 6 months old as older age children. Interestingly, Weisberg et al found no association between strabismus and age at cataract extraction [2].

In our study, we found esotropia to be common among all the strabismus-types post lensectomy. Similarly, David et al. showed a higher prevalence of esotropia [3]. Park et al and Weisberg et al found exotropia to be the most prevalent type of strabismus post-lensectomy in children [2, 6]. Park et al suggested that this higher prevalence of exotropia most likely was influenced by race since the study was conducted in South Korea, predominantly Asians [6]. On the other hand, France and Frank found nearly equal numbers of esotropia and exotropia in their study of aphakic children [7]. In our study, the reason for the development of strabismus in some of the children post-operation is still not well understood since the same management was offered for both strabismic and non-strabismic groups. Our findings deepen the current understanding of strabismus associated with lensectomy in infants. This study also makes some important contributions to visual outcomes in infants managed with RGP contact lenses post unilateral or bilateral lensectomy. 

Study design and small sample size are important limitations. This could have led to an overestimation of the outcome of visual acuity and the development of strabismus in children who had unilateral cataract surgery. The retrospective design may introduce bias, and the variability in surgical approaches (different surgeons, techniques) could affect outcomes. Future research should reconfirm these findings by conducting larger-scale prospective studies.

## Conclusions

Strabismus, most commonly esotropia, is common after congenital lensectomy, particularly in those children who underwent unilateral cataract surgery, and in those less than 6 months of age. Children undergoing unilateral cataract surgery, especially at an early age, should be followed up carefully for the development of strabismus. Understanding the interocular visual acuity of infants and the frequency of occurrence of strabismus post cataract surgery is of great essence as this will create awareness for them to be followed up carefully for the development of strabismus and early treatment of amblyopia to prevent permanent vision loss. Further research is needed to better understand the mechanism of strabismus and vision loss in this population.
